# Method development to reduce the fiber content of wheat bran and rice bran through anaerobic fermentation with rumen liquor for use in poultry feed

**DOI:** 10.5713/ajas.18.0446

**Published:** 2018-09-13

**Authors:** Momota Rani Debi, Brigitta A Wichert, Annette Liesegang

**Affiliations:** 1 Institute of Animal Nutrition, Vetsuisse Faculty, University of Zurich, Winterthurerstrasse 270, 8057 Zurich, Switzerland

**Keywords:** Fibrous Feed, Two-step Fermentation, Nutritive Value, Poultry Nutrition, Developing Countries

## Abstract

**Objective:**

Wheat bran (WB) and rice bran (RB) are the agricultural by-products used as poultry feed in many developing countries. However, their use for poultry feed is limited due to high fiber and the presence of anti-nutritional substances (e.g. β-glucans). The objective of this study was to develop a method to improve the quality of those brans by reducing the fiber content.

**Methods:**

A two-step fermentation method was developed where the second fermentation of first fermented dry bran was carried out. Fermentation was performed at a controlled environment for 3 h and 6 h (n = 6). The composition of brans, buffer solution and rumen liquor was maintained in a ratio of 1:2:3, respectively. Brans were analyzed for dry matter, crude fiber (CF), acid detergent fiber (ADF), neutral detergent fiber (NDF), and acid detergent lignin (ADL) content. Celluloses and hemicelluloses were calculated from the difference of ADF-ADL and NDF-ADF, respectively. Samples were compared by two-factor analysis of variance followed by Tukey’s multiple comparison tests (p<0.05).

**Results:**

CF %, ADF % and cellulose tended to decrease and NDF % and hemicellulose content was reduced significantly (p<0.05). After the 1st fermentation step, NDF decreased 10.7%± 0.55% after 3 h vs 17.0%±0.78% after 6 h in case of WB. Whereas, these values were 2.3%± 0.30% (3 h) and 7.5%±0.69% (6 h) in case of RB. However, after the 2nd fermentation step, the decrease in the NDF content amounted to 9.1%±0.72% (3 h), 17.4%±1.13% (6 h) and 9.3%±0.46% (3 h), 10.0%±0.68% (6 h) in WB and RB, respectively. Cellulose and hemicellulose content was reduced up to 15.6%±0.85% (WB), 15.8%±2.20% (RB) and 36.6%±2.42% (WB), 15.9%±3.53% (RB), respectively after 2nd fermentation of 6 h.

**Conclusion:**

Two-step fermentation process improved the quality of the brans for their use in poultry feed.

## INTRODUCTION

In developing countries, poultry industry plays a vital role to meet the protein requirements for human nutrition. For this reason, the production efficiency of poultry to convert feed into meat is essential [1,2]. The problem is that quality poultry feed with reasonable prices are lacking and low production is the result in developing countries [3]. Maize and soybean meal are the major ingredients as energy and protein sources, respectively. But also human beings in developing countries consume different grains and soybean to cover their daily nutritional requirements [2]. As a consequence, to reduce the feed costs and to overcome the unavailability of quality poultry feed, some low quality agricultural by-products are used along with main ingredients [3]. Among the available sources, wheat bran (WB) and rice bran (RB) are the abundant and cheap agricultural by-products in rice and wheat producing countries [4] that are used as components in poultry feed [3]. Although these are inexpensive, high fiber content limits their use in poultry feed as the high fiber content reduces the digestibility of nutrients [5]. In addition, brans also contain some anti-nutritional substances e.g. β-glucans [6]. In RB and WB, the neutral detergent fiber (NDF) content varies from 33% to 40% and 48% to 51%, respectively [7]. β-Glucans adversely affects the utilization of other nutrients, especially protein and starch utilization and produce highly viscous conditions in the small intestine of the chicken [8] that contribute to delayed gastrointestinal absorption. On the other hand, the digestive system of poultry is not capable to produce enzymes to hydrolyze non-starch polysaccharides [5] present in brans. Therefore, the availability of nutrients is reduced if poultry consumes these fibrous feed and that depresses the production performance [4]. Different exogenous feed enzymes have been used in poultry diet to increase the digestibility of nutrients [9]. However, the use of specific enzyme is costly in one hand and on the other hand, the effectiveness of these enzymes depends on many factors. One of the most important factor is pH and the catalytic activity of different enzymes depends on the pH level. A varied range of pH in the digestive tract of poultry becomes the first physiological limitation for the activity and stability of exogenous enzymes [10]. Additionally, the feed spends relatively short time in the digestive tract of poultry which is another limiting factor for ineffectiveness of exogenous feed enzymes [10]. For these reasons, it is very important to reduce the fiber content of the brans by any processing method. In this regard, fermentation with rumen liquor (containing microbes) could be an effective method to overcome these problems. Rumen microbes can synthesize β-glucanases, cellulases, and hemicellulases the enzymes required for the breakdown of cellulose, hemicelluloses and phenolic polymers [11,12]. Fiber is degraded by a combination of ruminal bacteria, fungi and protozoa [11], where approximately 80% of this degradation performed by bacteria and fungi and 20% by protozoa [13]. To the author’s best knowledge, the use of specific fibrolytic enzymes or microbes are common methods of fermentation but limited data are available on fermentation with rumen liquor by maintaining appropriate environment for microbes to decrease the fiber content to produce high quality feed components for poultry.

First some experiments (single-step fermentation) were performed to find optimum fermentation conditions. Fermentation of WB was carried out in different composed mixtures under different environmental conditions. Fermentation was started with 48 h then reduced gradually (48, 36, 24, 12, 6, and 3 h) to 3 h due to the instability of the pH for fiber degrading bacteria. It was found that, optimum pH (6.5) was maintained up to 3 h then decreased to 5.1, 4.7, 4.2, 4.0, 3.9, and 3.8 after 6, 8, 12, 24, 36, and 48 hours, respectively. As optimum pH could not be maintained, further nutrient analyses were not performed. But few samples (from 3 h fermentation) when analyzed it was observed that, after 3 h the fiber content did not decrease satisfactorily then a method with two-step fermentation was performed.

It would be feasible to implement the developed method at the industrial scale production. The new fermentation technology will be of great benefit and would probably be provided to the rice/wheat miller for the production of fermented brans in a large scale to introduce a quality product to the feed market for economic poultry ration formulation. It might also be possible to use this technology to ferment other kinds of fibrous feed for feeding other non-ruminants. A designed fermenter would be useful for industrial scale production and a fermenter might be designed to facilitate all the conditions suitable for proper growth of rumen microorganisms. In developing countries, it is promising to collect rumen liquor as well as rumen content from different slaughtered cows and goats that would be a good sources of inoculum. It might be useful to reuse the effluent after fermentation as a inoculum source for large scale production. It might also be feasible to grow rumen bacteria in a laboratory that might be useable for large scale fermented feed production.

Considering all these factors, the present study (two-step fermentation) was undertaken to systematically evaluate and compare the effect of fermentation on nutritional improvement of WB and RB after fermentation with rumen liquor. The hypothesis is that, fermentation of different kinds of brans with rumen liquor will improve their nutritive value by decreasing the fiber content. Thus the aim of this present study was to develop an easy and cheap method for optimum fermentation of high fiber feedstuffs to improve their nutritive value due to lower fiber content.

## MATERIALS AND METHODS

### Part 1: Fermentation of wheat bran

All experiments (fermentation and nutrient analysis) were performed at the laboratory of the Institute of Animal Nutrition, Vetsuisse faculty, University of Zurich, Switzerland according to the animal welfare law of Switzerland.

#### Preparation of buffer solution

McDougall buffer [14] solution (5,000 mL) was prepared by mixing ‘Solution A’ (50 mL) with ‘Solution B’ (4,950 mL), where the composition of this solution is given in Table 1. The pH of that buffer solution was 8.1 to 8.2 and pH measurement was performed with a digital pH meter (827 pH lab, Metrohm AG, Herisau, Switzerland).

#### Collection and evaluation of rumen liquor

Rumen liquor was obtained from a cannulated Brown Swiss cow, Department for Farm Animals, Vetsuisse Faculty, University of Zurich, Switzerland that was fed with hay and concentrates for maintenance. Rumen liquor collection was done before morning feeding [15] in a previously warmed (39°C) [16,17] insulated flask that was flashed with CO_2_ gas before collection to maintain the anaerobic condition. After collection, the rumen liquor was filtered through a stainless steel round strainer to remove any solid particle present. After that the filtered rumen liquor was transferred immediately (within 5 minutes) into fermentation containers with constant flushing of CO_2_ gas to maintain anaerobic condition [17]. Simultaneously, pH and temperature of the rumen liquor were measured immediately following collection. The pH of the collected rumen liquor was within a range from 6.4 to 7.3 during the whole experiment and temperature was between 37°C to 40°C. Physical characteristics such as color, odor, and consistency were recorded and a methylene blue reduction test (MBRT) was carried out to assess the amount of functional anaerobic bacteria available in the rumen liquor according to the procedure described by DePeters and George [18]. Only if the measurements indicated that collected rumen liquor was normal with an adequate amount of microbes, the fermentation was started.

#### Preparation of fermentation mixture and fermentation (Two-step fermentation)

##### i) 3 h fermentationi)

**Figure 1:**
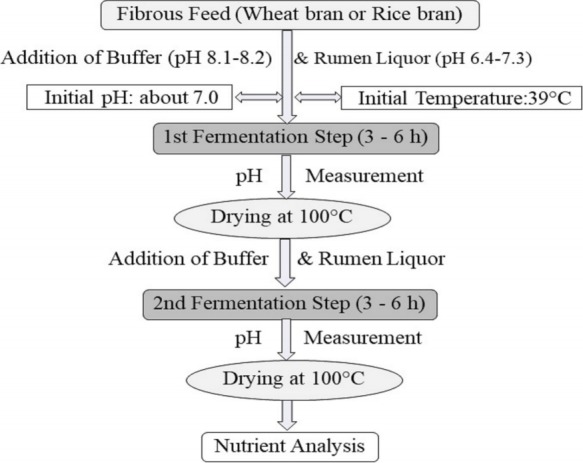
The design of the two step fermentation procedure of wheat bran (WB) and rice bran (RB) with rumen liquor for 3 h and 6 h.

Due to the results of pH and crude nutrient measurements after the single-step fermentation, a two-step fermentation was tested. In the two-step fermentation, a 2nd fermentation of the 1st fermented bran after drying of the mixture from the first fermentation (1st step) was performed (Figure 1). For the second fermentation step, buffer solution and rumen liquor were added again following the same procedure as in the 1st fermentation step. In that case, fermentation of WB was carried out in an Erlenmeyer flask fitted with a gas bag at 39°C for 3 h. The composition of the fermentation mixture was maintained in a ratio of 1:2:3 for brans, buffer solution [14] and rumen liquor, respectively in both fermentation steps. After the 1st fermentation step, the mixtures were dried in an oven at 100°C and then the fermentation procedure started again (2nd step of fermentation) with this dry fermented bran. After the 2nd fermentation step, the remaining material was dried again at 100°C. The pH was measured as described in the pH measurement schedule (Table 2). Optimum temperature (39°C), pH (6 to 6.5) as well as anaerobic condition was maintained during both steps of fermentation. Time of 1st and 2nd fermentation step was the same. Each fermentation step was carried out with 6 samples (n = 6) at 6 different days.

##### ii) 6 h fermentation

Another experimental protocol that was tested was a 6 h fermentation with an additional buffer substance NaHCO_3_ (1% level of total mixture weight) that was added after 3 h to maintain an appropriate pH for the entire period of fermentation. This was done because after 3 h, the pH started to decrease below 6 in the 3 h fermentation process. Due to this fact, at that point an additional buffer substance was added to increase the pH near to 7. The same procedure was used in both steps of fermentation. Composition mixture of fermentation and experimental procedure was similar in the 6 h like in the 3 h fermentation. The pH was measured as described in the pH measurement schedule (Table 2).

##### Collection of samples

Samples were taken from different stages (5 stages) of this two-step fermentation process for nutrient analysis: stage 1, fresh bran; stage 2, bran before 1st fermentation step, after addition of rumen liquor with buffer and bran mixture; stage 3, 1st fermented dried bran after 1st fermentation step; stage 4, fermentation product mixture (1st fermented product+buffer+rumen liquor), before 2nd fermentation step; and stage 5, 2nd fermented dried bran after 2nd fermentation step.

##### Nutrient analysis

All samples were analyzed for crude fiber (CF, proximate analysis) and van Soest fibers [19] according to the VDLUFA method book III [20]. DM was analyzed from fresh as well as dry fermented samples by drying at 105°C in a compartment dryer (Binder FED 53-UL Laboratory compartment dryer) for 3 h until weight constancy. Duplicates of each sample were analyzed and mean values were calculated. Celluloses and hemicelluloses were calculated from the difference of acid detergent fiber (ADF)-acid detergent lignin (ADL) and NDF-ADF respectively [21,19] to know how much the fiber content actually decreased during the two-step fermentation process. Changes in nutrient content are given as percentage (%).

### Part 2: Fermentation of rice bran

The experimental procedure was similar as in WB fermentation. The only difference was in the 6 h fermentation. During the 2nd step of the 6 h fermentation, no additional buffer substances were added as pH was stable enough (pH: 7) in that case.

### Statistical analysis

Statistical analyses were performed using the statistical software package IBM SPSS, version 23 (IBM SPSS Statistics for Windows 2015, IBM Corp, New York, USA). All nutrients data were analyzed by two-way analysis of variance (ANOVA) followed by Tukey’s multiple comparison tests (p<0.05). Factors in the analyses were time and different stages of fermentation. The pH measurement data were analyzed by one-way ANOVA for 3 h and 6 h separately, as pH measurement stages were different between 3 h and 6 h fermentation. The results are given as mean±standard error of mean.

## RESULTS

### Part 1

#### pH

##### i) 3 h fermentationi)

**Figure 2:**
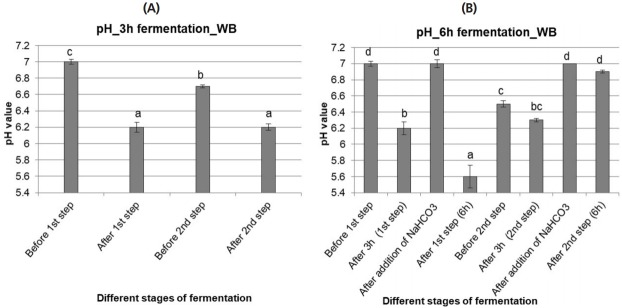
pH values of wheat bran (WB) fermented with rumen liquor for 3 h (A) and 6 h (B). The values with different letters differ significantly at p<0.05 level (Tukey’s honestly significant difference). Values are mean±standard error of mean; n = 6.

During 3 h fermentation, a pH of 6 to 6.5 was maintained in both fermentation steps and the pH was decreased significantly (p<0.05) from the initial value of 7 in both of the steps (Figure 2A).

##### ii)6 h fermentation

The 6 h fermentation was started at pH 7 (7.0±0.03) like the 3 h fermentation. The pH was significantly (p<0.05) reduced after fermentation (Figure 2B). After the 1st fermentation step, pH was 5.6±0.14. In the 2nd fermentation step, the pH was not reduced as much as after the 1st step (pH 6.9±0.02).

#### Fiber content

##### i) Neutral detergent fiber %

The NDF content of WB was significantly (p<0.05) reduced after fermentation during the 1st (stage 2 to 3) and 2nd (stage 4 to 5) step of fermentation for both 3 h (p = 0.000 for both 1st and 2nd step) and 6 h (p = 0.000 for both 1st and 2nd step) fermentation (Table 3). In that case, fermentation time had a significant effect on the NDF content and a significant (p = 0.001) difference was observed between 3 h and 6 h fermentation during the 2nd fermentation step. In the 1st fermentation step, NDF was reduced more during 6 h compared to 3 h fermentation but no significant difference was observed. Data on percent changes are given in Supplementary Table S1.

##### Crude fiber, acid detergent fiber, and acid detergent lignin %

Analyses of the CF and ADF content data showed no significant (p<0.05) reduction of these nutrients after fermentation except in the 2nd step of the 6 h fermentation, where CF was significantly reduced (p = 0.001) (Table 3). Only a decreasing trend of the CF and ADF content was observed during each step for both the 3 h and 6 h fermentation without any time difference. Detailed data on percent changes are given in Supplementary Table S1. Here it could be shown that CF and ADF decreased more during 6 h of fermentation compared to 3 h. Data also revealed that there were no significant (p>0.05) differences of ADL content after the 1st step (stage 2 to 3) and 2nd step (stage 4 to 5) of fermentation for both 3 h and 6 h fermentation (Table 3).

##### Cellulose and hemicellulose %

**Figure 3:**
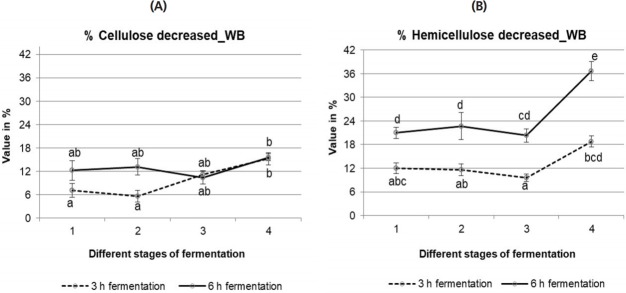
Content (%) of cellulose (A) and hemicellulose (B) reduction of wheat bran (WB) fermented with rumen liquor for 3 h and 6 h. Stage 1, during 1st fermentation step; Stage 2, during 2nd fermentation step; Stage 3, fresh bran to 1st fermented bran; Stage 4, fresh bran to 2nd fermented bran. The values with different letters differ significantly at p<0.05 level (Tukey’s honestly significant difference). Values are mean±standard error of mean; n = 6.

Data are presented in Figure 3A and 3B for cellulose and hemicellulose reduction, respectively. Cellulose and hemicellulose were significantly (p<0.05) influenced by the two-step fermentation method. Specifically, the effect of time on the reduction of hemicellulose content was significant (p<0.05) during both steps of fermentation. Hemicellulose was significantly more reduced (p<0.05) after 6 h compared to 3 h fermentation. However, in the case of cellulose content, no significant difference was found between 3 h and 6 h fermentation.

### Part 2

#### pH

##### i) 3 h fermentationi)

**Figure 4:**
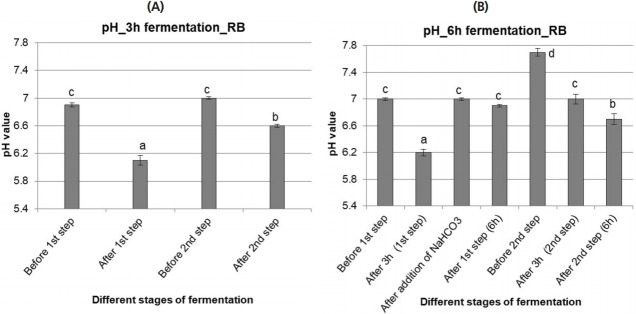
pH values of rice bran (RB) fermented with rumen liquor for 3 h (A) and 6 h (B). The values with different letters differ significantly at p<0.05 level (Tukey’s honestly significant difference). Values are mean±standard error of mean; n = 6.

The pH measurement data of 3 h fermentation is presented in Figure 4A. During 3 h fermentation, an optimum pH (>6) was maintained in both steps of RB fermentation. Nevertheless, the pH was reduced significantly (p< 0.05) during both steps of fermentation.

##### ii) 6 h fermentation

Data are presented in Figure 4B. In case of the 1st step of RB fermentation, the pH was similar as after WB fermentation during first 3 h. However, the pH did not decrease so much while the 1st fermentation step was stopped. In the 2nd fermentation step, the pH was stable without NaHCO_3_ up to the end of the process after 6 h.

#### Fiber content

##### i) Neutral detergent fiber %

The effects of fermentation on NDF content are presented in Table 4. The NDF content was reduced significantly (p<0.05) by two-step fermentation. During the 6 h fermentation, a significant reduction was found in both steps of fermentation (1st step: p = 0.002) and 2nd step: p = 0.000). However, in case of 3 h fermentation, a significant reduction was observed during the 2nd step of fermentation only. Detailed data on percent changes are given in Supplementary Table S1.

##### ii) Crude fiber, acid detergent fiber, and acid detergent lignin %

Data of CF, ADF, and ADL content are presented in Table 4. CF content was reduced significantly (p = 0.000) only in the 2nd step of the 6 h fermentation. In case of ADF content, there was a significant reduction observed in the 2nd step of both 3 h (p = 0.006) and 6 h (p = 0.003) fermentation. There was no significant difference observed in case of ADL content during both 3 h, 6 h, and 1st and 2nd step of fermentation.

##### iii) Cellulose and hemicellulose %

**Figure 5:**
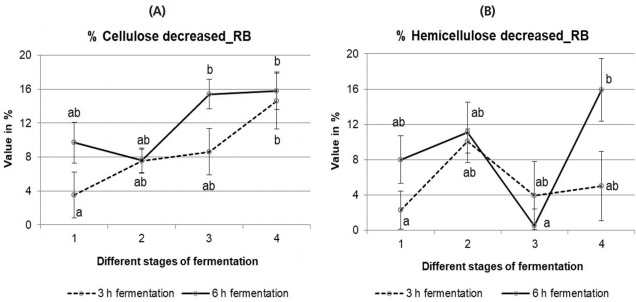
Content (%) of cellulose (A) and hemicellulose (B) reduction of rice bran (RB) fermented with rumen liquor for 3 h and 6 h. Stage 1, during 1st fermentation step; Stage 2, during 2nd fermentation step; Stage 3, fresh bran to 1st fermented bran; Stage 4, fresh bran to 2nd fermented bran. The values with different letters differ significantly at p<0.05 level (Tukey’s honestly significant difference). Values are mean±standard error of mean; n = 6.

Percentages of cellulose and hemicellulose reduction of fresh bran as well as fermented RB are presented in Figure 5A and 5B, respectively. No significant difference was observed in cellulose reduction during RB fermentation. Hemicellulose was reduced more after 6 h compared to 3 h fermentation and a significant (p = 0.015) lower hemicellulose content was measured in the 2nd fermented bran compared to the 1st fermented bran after 6 h fermentation.

## DISCUSSION

In the present investigation, a two-step fermentation was performed after examining the results of some initial experiments (single-step fermentation) of this study that were conducted based on some in-vitro studies [22,23] such as rumen simulation technique (RUSITEC). However, RUSITEC systems are not useful for commercial feeding purpose or industrial use. A major problem to continue the single-step (initial experiments) fermentation for a longer period of time is the decrease of pH of the fermented mixture with increasing the time of fermentation. Due to these facts, the single-step fermentation was not continued. However, it seemed to be necessary to increase the fermentation time to increase the chances of microbe growth and with this the production of fiber degrading enzymes [11]. To overcome these problems, a two-step fermentation method was developed. It was assumed that most of the components that require less time to degrade, would be fermented during the 1st step of fermentation and that the amount of fiber would be reduced in the 2nd fermentation step. The 1st fermentation step was stopped when the pH decreased to a level of <6 that is known to be incompatible with the growth of fiber degrading bacteria.

The pH is one of the most important factors for ruminal microorganisms for the fermentation of fibrous components [24,25]. The appropriate range of rumen pH should be between 6 and 7 if cow maintain with proper ratio of grass and concentrate with good physiological conditions and a pH lower than that decreases the extent of fiber degradation process [26,27]. In the present study, the pH was reduced significantly after each step of fermentation and continued to decrease with increasing fermentation time. Also rumen pH (in live animal) changes constantly and depends on several factors e.g. the production of saliva, the generation and absorption of volatile fatty acids (VFA), the type and level of feed intake, and the exchange of buffer through the ruminal epithelium [28]. In the used in-vitro system most likely the pH was reduced due to the formation of VFA [27–29] and lactic acids [27,28] during fermentation and accumulation of these acids [30]. As a weak acid, these organic acids rapidly dissociate and release a proton and thereby decrease pH under most circumstances. As our fermentation system was completely closed, absorption or removal of VFA or other acidic components were not possible. For this reason, additional buffer substances NaHCO_3_ were added to increase the pH and with this the fermentation capability of the system. As an appropriate pH (6 to 6.5) could maintained only during the 3 h fermentation, the additional buffer substance was added to control pH during the 6 h fermentation. NaHCO_3_ is a potent buffer to prevent acidic conditions in the rumen or used in in-vitro system [31,24] and with this produce appropriate fermentation conditions. Although the procedure used for fermentation was the same in RB as in WB, different pH values from WB to RB were measured during the whole fermentation process. This might be due to the compositional variation of WB and RB. At the end of the 2nd step of 6 h fermentation, pH was not as much reduced as after the 1st step and remained near to 7. This may indicate that most of the components of brans that degraded quickly were fermented during the 1st fermentation step. In future, the question should be answered if a longer 2nd fermentation would be useful to improve the nutritional value of the brans.

In the described fermentation system, fiber was degraded more during 6 h compared to 3 h fermentation and the fiber was decreased more in the 2nd fermentation step. This could be expected as fermentation time is a very important factor for fiber degradation and Wizna et al [32] reported that the fermentation time had a greater influence than the inoculum dose. Longer fermentation time at ideal temperature and pH condition means a higher amount of microbes within the fermentation process. A too short fermentation time might be the reason for the slow degradation of fiber during the 1st step of fermentation and also in the 3 h fermentation compared to 2nd step and 6 h fermentation. Jazi et al [33] reported that fermentation of cotton seed meal with *Bacillus subtilis*, *Aspergillus niger* and *A. oryzae* for 7 days significantly (p<0.05) reduced CF (34.73%, from 12.58% to 8.21%). Also during fermentation of RB with *Bacillus amyloliquefaciens* and humic substances for 3 and 5 days, CF was reduced from 10.62% to 8.37% and 8.36%, respectively [4]. Conversely, Aanuoluwapo and Gbenga [34] found that CF increased from 6.25% to 6.89% when WB was fermented with rumen liquor where the mixture of WB and rumen liquor were put in a polythene bags and kept under the soil for 3 days. They did not mention anything about the pH and temperature of the fermented mixture that are very important determinants for cellulose degrading bacteria. In the present investigation, the appropriate environments (pH, temperature, anaerobic condition) were maintained during the whole fermentation period. In this study, NDF content was clearly reduced in the 2nd fermented WB after 6 h compared to fresh bran. The fiber reduction was higher in WB compared to RB. Probably, the reasons for these differences are the different compositions of WB and RB. RB contains less crude protein (CP) than WB. Previous studies reported that cellulolytic bacteria increased significantly (p<0.05) with increasing level of CP content in the diet whereby increased fiber degradability [35,36]. Also in beef cattle and dairy cattle in other studies [37,38], the NDF degradability tended to increase with increasing dietary CP levels. Another reason could be the higher crude fat content in RB compared to WB which ranges from 4.07% to 19.31% depending on the sources of collection [6]. This might be another reason for slower degradation of fiber in RB compared to WB. A high fat content coats the dietary fiber and therefore interferes with the fiber degradation [39,40]. Additionally, RB has more lignification than WB. Due to the presence of strong covalent bonds between lignin and the cell wall polysaccharides, it reduces the accessibility for the enzymatic hydrolysis secreted by rumen microbes [41] and thereby reduces fiber degradation. In the present study, the lignin content was not changed significantly by fermentation because of rumen microbes can not synthesize lignin degrading enzymes. This is in agreement with van Soest [41]. However, it was observed that the ADL content of fermented bran was higher compared to fresh bran after both 1st and 2nd fermentation step. The reason for this could be a relative increase of lignin as other fiber components were degraded during fermentation and with this the ADL content that not degraded by fermentation, increased relatively [41].

The results obtained in this study suggest that fermentation of WB and RB with rumen liquor improved their nutritional value by decreasing the fiber content. Fiber degradation was strongly influenced by the time of fermentation and a 2nd fermentation step had a significant effect on fiber reduction compared to the 1st fermentation step. Further the procedure of fermentation should be adjusted according to the fermented feed-stuffs. In all *in-vitro* fermentation of feed stuffs to improve their nutritive value seems to be an easy but effective method. However, in future, the method should be optimized by a longer fermentation time and the improvement of nutritive value, especially the changes of amino acid (AA) profile should be investigated. Additionally, the method should be adapted for an industrial use for the improvement of the nutritional quality of different kinds of fibrous feed to be able to produce meat at affordable price. This would help to provide humans in the third world with AAs in a sufficient amount.

## Supplementary Data



## Figures and Tables

**Table 1:** Chemical composition of McDougall (1948) buffer solution (5,000 mL)

Constituents	Amount
Solution-A	
NaCl (g)	2.35
KCl (g)	2.85
CaCl_2_.2H_2_O (g)	0.27
MgCl_2_.6H_2_O (g)	0.64
Distilled water (mL)	50
Solution-B	
NaHCO_3_ (g)	49.00
Na_2_HPO_4_ (g)	18.625
Distilled water (mL)	4,950

**Table 2:** pH measurement schedule for 3 h and 6 h fermentation of wheat bran and rice bran

Parameter	Fermentation	

	3 h	6 h
Collected rumen liquor	×	×
Before 1st step	×	×
After 3 h of 1st step	×	×
After addition of NaHCO_3_	-	×
After 6 h of 1st step	-	×
Before 2nd step	×	×
After 3 h of 2nd step	×	×
After addition of NaHCO_3_	-	×
After 6 h of 2nd step	-	×

×, pH was measured.

**Table 3:** Fiber fractions (%) of wheat bran (WB) fermented with rumen liquor for 3 h and 6 h

Items	Different stages of fermentation1)				

	1	2	3	4	5
3 h fermentation					
NDF %	48.77^de^±0.32	49.79^e^±0.85	44.47^bc^^*^±0.49	46.49^cd^±0.82	42.26^b^^*^±0.15
CF %	9.82abc±0.10	10.08^bc^±0.18	9.55abc±0.16	9.86abc±0.14	9.39^ab^±0.09
ADF%	13.88^ab^±0.21	13.88^ab^±0.91	12.94^a^±0.18	14.41^bc^^*^±0.18	13.95abc±0.25
ADL %	3.99^a^±0.07	4.43^a^±0.16	4.16^a^±0.07	5.53^b^^*^±0.17	5.57^b^^*^±0.21
6 h fermentation					
NDF %	49.60^e^±0.36	50.81^e^±0.71	42.19^d^^*^±0.52	46.20^cd^±0.74	38.17^a^^*^±0.71
CF %	9.87abc±0.12	10.33^c^±0.25	9.60abc±0.18	10.27^c^±0.13	9.13^a^±0.16
ADF%	13.48^ab^±0.27	14.36^bc^±0.42	13.40^ab^±0.27	16.52^d^^*^±0.37	15.28^cd^±0.38
ADL %	4.14^a^±0.13	4.81^ab^±0.16	5.05^ab^±0.21	7.44^c^^*^±0.38	7.40^c^^*^±0.43

NDF, neutral detergent fiber; CF, crude fiber; ADF, acid detergent fiber; ADL, acid detergent lignin.

1) Stage 1, fresh WB; Stage 2, bran before 1st fermentation step after addition of rumen liquor and buffer; Stage 3, bran after 1st fermentation step (1st fermented dried WB); Stage 4, bran before 2nd fermentation step after addition of rumen liquor and buffer with 1st fermented dried WB; Stage 5, bran after 2nd fermentation step (2nd fermented dried WB).

a–e Significant (p<0.05 level; Tukey’s honestly significant difference) differences between stages are given in lines by different small superscripts. Significant differences between fermentation hours at one stage are given by ^*^.

Values are mean±standard error of mean; n = 6.

**Table 4:** Fiber fractions (%) of rice bran (RB) fermented with rumen liquor for 3 h and 6 h

Items	Different stages of fermentation1)				

	1	2	3	4	5
3 h fermentation					
NDF %	40.28^cd^±0.55	39.67bcd±0.43	38.75^bc^±0.54	41.24^d^±0.29	37.41^ab^±0.56
CF %	15.49^ab^±0.26	15.80abc±0.20	16.40^bc^±0.16	16.53^b^^*^±0.28	15.56abc±0.12
ADF%	18.91^c^±0.48	18.70^bc^±0.34	18.30abc±0.12	18.80^c^±0.19	17.23^a^±0.26
ADL %	6.92^a^±0.04	7.36^ab^±0.13	7.39^ab^±0.12	7.80^bc^±0.11	7.06^ab^±0.20
6 h fermentation					
NDF %	39.98^cd^±0.26	41.23^d^±0.33	38.16^bc^±0.65	39.19bcd±0.46	35.27^a^±0.62
CF %	15.70abc±0.12	15.80abc±0.13	15.49^ab^±0.22	16.57^c^^*^±0.38	15.05^a^±0.15
ADF%	18.73^c^±0.16	18.32abc±0.28	17.09^a^±0.18	19.08^c^±0.33	17.43^ab^±0.15
ADL %	6.93^a^±0.06	7.26^ab^±0.14	7.11^a^±0.11	8.34^c^±0.23	7.51^bc^±0.07

NDF, neutral detergent fiber; CF, crude fiber; ADF, acid detergent fiber; ADL, acid detergent lignin.

1) Stage 1, fresh RB; Stage 2, bran before 1st fermentation step after addition of rumen liquor and buffer; Stage 3, bran after 1st fermentation step (1st fermented dried RB); Stage 4, bran before 2nd fermentation step after addition of rumen liquor and buffer with 1st fermented dried RB; Stage 5, bran after 2nd fermentation step (2nd fermented dried RB).

a–d Significant (p<0.05 level; Tukey’s honestly significant difference) differences between stages are given in lines by different small superscripts. Significant differences between fermentation hours at one stage are given by ^*^.

Values are mean±standard error of mean; n = 6.
