# Supplementary material for: Method development to reduce the fiber content of wheat bran and rice bran through anaerobic fermentation with rumen liquor for use in poultry feed
Source: Asian-Australas J Anim Sci. 2018 Sep 13;32(3):395–404. doi: 10.5713/ajas.18.0446 (PMC6409478; doi:10.5713/ajas.18.0446)
Supplement: Supplementary file 1 [file ajas-18-0446-supplementary.pdf]

Supplementary Information

Supplementary Table 1. Percent nutrient changes during two step fermentation of wheat bran (WB) and rice bran (RB) with rumen liquor for 3 h and 6 h. Values were calculated from the original data

Parameters		From stage 2-3 (1st fermentation step)		From stage 4-5 (2nd fermentation step)		From stage 1-3 (Fresh bran to 1st fermentation step)		From stage 1-5 (Fresh bran to 2nd fermentation step)	
		3 h	6 h	3 h	6 h	3 h	6 h	3 h	6 h
CF (%)	WB	5.3±0.09 (↓)	7.1±0.23 (↓)	4.8±0.12 (↓)	11.1±0.29 (↓)	2.8±0.06 (↓)	2.8 ± 0.10 (↓)	4.4±0.08 (↓)	7.5±0.08 (↓)
	RB	3.8±0.14 (↑)	2.0±0.22 (↓)	5.9±0.33 (↓)	9.2±0.52 (↓)	5.9±0.15 (↑)	1.3±0.18 (↓)	0.5±0.26 (↑)	4.1±0.23 (↓)
ADF (%)	WB	6.8±0.12 (↓)	6.7±0.27 (↓)	3.2±0.28 (↓)	7.5±0.19 (↓)	6.8±0.08 (↓)	0.6±0.15 (↓)	0.5±0.38 (↑)	13.4±0.60 (↑)
	RB	2.2±0.36 (↓)	6.7±0.42 (↓)	8.4±0.32 (↓)	8.7±0.39 (↓)	3.2±0.43 (↓)	8.8±0.33 (↓)	8.9±0.70 (↓)	6.9±0.29 (↓)
NDF (%)	WB	10.7±0.55 (↓)	17.0±0.78 (↓)	9.1 ±0.72 (↓)	17.4±1.13 (↓)	8.8 ±0.34 (↓)	15.0±0.70 (↓)	13.4±0.33 (↓)	23.1 ± 0.97 (↓)
	RB	2.3±0.30 (↓)	7.5±0.69 (↓)	9.3±0.46 (↓)	10.0±0.68 (↓)	3.8±0.73 (↓)	4.6±0.58 (↓)	7.1±0.91 (↓)	11.8±0.78 (↓)

Stage 1: Fresh bran Stage 2: bran before 1st fermentation step after addition of rumen liquor and buffer Stage 3: bran after 1st fermentation step (1st fermented dried bran) Stage 4: bran before 2nd fermentation step after addition of rumen liquor and buffer with 1st fermented dried bran Stage 5: bran after 2nd fermentation step (2nd fermented dried WB). The values are Mean of % change ± Standard error of mean; n = 6; ↑ = increased, ↓ = decreased.
